# Deep learning in the early diagnosis of acute aortic dissection

**DOI:** 10.3389/fcvm.2026.1828870

**Published:** 2026-06-12

**Authors:** Simon Eggleton, Jon Ryan, Blanca Gallego

**Affiliations:** 1 Centre for Big Data Research in Health, University of New South Wales, Sydney, NSW, Australia; 2 Department of Cardiology, Prince of Wales Hospital, Randwick, NSW, Australia; 3 Department of Cardiothoracic Surgery, Prince of Wales Hospital, Randwick, NSW, Australia; 4 Discipline of Surgery, University of New South Wales, Sydney, NSW, Australia

**Keywords:** acute aortic dissection, artificial intelligence, deep learning, diagnostic efficiency, diagnostic pathway, machine learning

## Abstract

This review explores the potential of deep learning (DL) to enhance the diagnostic efficiency of acute aortic dissection (AAD), a life-threatening cardiovascular emergency characterised by high misdiagnosis rates. In contrast to a prior systematic review that evaluated DL approaches to aid diagnosis, this review focuses specifically on the early diagnostic phase, examining routinely available investigations, including the electrocardiogram, chest x-ray and tabular clinical data, to inform decisions regarding which patients should and should not proceed to advanced imaging. We analyse current diagnostic challenges contributing to AAD misdiagnosis and existing diagnostic decision support pathways. The review also examines healthcare data modalities and DL architectures capable of identifying complex patterns and relationships not readily recognised by clinicians. We summarise existing DL research in the early phase of AAD diagnosis aimed at improving diagnostic accuracy and offer insights into future research directions. Finally, challenges relevant to DL-based AAD diagnosis are discussed, including data scarcity, class imbalance due to its low prevalence, and the role of explainable AI.

## Introduction

Acute aortic dissection (AAD) is a cardiovascular emergency characterised by a tear in the inner layer of the aorta, resulting in blood entering the wall, creating a pressurised false lumen which can propagate and rupture the vessel (1). Untreated, the mortality of an acute dissection of the ascending aorta at 1 day is reported as 47.3% (2), highlighting the importance of early diagnosis to facilitate prompt initiation of treatment.

Computed tomography aortography (CTA), a form of advanced imaging, is commonly used to diagnose AAD. However, the low prevalence of AAD among patients presenting with suspicious symptoms results in a low diagnostic yield (3, 4).

A systematic review found the misdiagnosis rate of AAD was 33.8% (5). Furthermore, patients with AAD misdiagnosed as having acute coronary syndrome (ACS) are typically administered antithrombotic therapy which increases bleeding, the requirement for blood products, and may increase mortality (6).

Given many patients undergo a diagnostic investigation with risks of contrast nephropathy and ionising radiation without having an AAD, while others with AAD are misdiagnosed, there is a need to improve the diagnostic efficiency in AAD.

Machine learning (ML) can process large volumes of data, identifying relationships to make predictions. Deep learning (DL), a subset of ML, uses artificial neural networks with multiple hidden layers to automatically extract features and learn complex relationships potentially imperceptible to humans. In patients presenting with chest pain, clinicians commonly integrate and appraise tabular data, electrocardiograms (ECG) and chest x-rays (CXR) to make clinical decisions. This process requires time and expertise, and subtle relationships may not be identified. Applying DL techniques, particularly within a multimodal framework, may improve the identification of patients suspected of an AAD who require advanced imaging.

This narrative review explores current research applying DL techniques for the diagnosis of AAD, with a specific focus on the early diagnostic phase prior to advanced imaging. Unlike a prior systematic review that evaluated DL performance across all stages of AAD diagnosis, this review concentrates on models utilising routinely available clinical data including the ECG, CXR, and tabular data, to support clinical decisions and improve diagnostic efficiency. The aim is to identify how DL may assist in determining which patients should, and should not, undergo advanced imaging. The review begins by outlining AAD, current diagnostic strategies, and their limitations, before transitioning to DL applications, their advantages and challenges, and finally future research opportunities.

## Acute aortic dissection

Acute aortic dissection results from an intimal tear forming a pressurised false lumen. This can disrupt the aortic valve with subsequent valvular regurgitation, obstruct branch vessels with organ malperfusion, and rupture the vessel resulting in catastrophic haemorrhaging.

Several systems serve to classify AAD. The Stanford classification is the most widely used AAD classification system as its anatomical descriptors align with treatment strategies. Type A involves the ascending aorta whilst type B is limited to the descending aorta (7). Open surgical repair is typically first-line therapy for type A, whilst medical therapy, and more recently endovascular repair in complicated cases are first-line treatment for type B (8).

In Australia, the incidence of AAD is 3.5 per 100,000 person-years. However, this figure likely underestimates its true incidence (9). Acute aortic syndromes, of which AAD is one form, have the highest incidence of death and disability-adjusted life years lost among all surgical emergencies (10).

The development of AAD is an interplay between various factors including age, hypertension, genetic predisposition, inflammation and atherosclerosis (11). The risk of AAD increases with aortic diameter, rising from 2% per year for diameters <50 mm to 6.9% when >60 mm (12).

### Presentation

Patients most commonly present with sudden-onset severe chest or back pain (13). However, in some patients, symptoms and signs of the sequelae of AAD dominate. For instance: hypotension secondary to cardiac tamponade or haemorrhage; heart failure from acute aortic valve regurgitation; or organ malperfusion or limb ischaemia due to arterial obstruction. This broad spectrum of symptoms can lead clinicians to consider alternative aetiologies.

### Diagnostic strategies

The diagnostic pathway for AAD is typically the same for any patient with chest pain. It begins with a history, examination, 12-lead ECG, CXR and serum biomarkers including cardiac troponin and often the fibrinogen degradation product D-dimer. Based on clinical suspicion and initial investigations, a decision regarding the need for advanced imaging is made.

Advanced imaging includes CT aortography, transoesophageal echocardiography and magnetic resonance aortography, which are considered the diagnostic investigations of choice to rule in and rule out AAD. Their performance is comparable, with CT aortography generally more accessible and with the highest negative likelihood ratio (14).

However, the diagnosis of AAD is not straightforward. AAD accounts for only 1 in 980 atraumatic chest pain ED presentations (15). It can mimic other conditions through involvement of the coronary ostia resulting in coronary ischaemia indistinguishable from plaque rupture, or stroke from carotid or brachiocephalic artery involvement. Additionally, early investigations are neither highly sensitive nor specific. These factors result in a reported AAD misdiagnosis rate of 33.8%, and patients without malperfusion who encounter diagnostic delay have a 10.5% absolute increase in 30-day mortality (16).

Diagnostic decision support pathways (DDSP) have been introduced to improve the diagnostic efficiency of AAD. In 2010 the American Heart Association and American College of Cardiology published an algorithm for evaluating patients suspected of AAD. This became the basis of the Aortic Dissection Detection Risk Score (ADD-RS), a DDSP incorporating predisposing conditions, chest pain description and high-risk examination features. A score of 0 is considered low-risk and a score of 2 or 3 is considered high-risk, at which point advanced imaging is recommended (17). A retrospective validation study in 2011 found an ADD-RS of >0 had strong sensitivity of 95.7% with uncertain specificity (18). In a subsequent observational cohort study with 0.3% AAD prevalence, the ADD-RS achieved moderate discriminatory performance with an Area Under the Receiver Operator Characteristic curve (AUROC) of 0.68 (4).

D-dimer has since been identified as an adjunct which improves ADD-RS's sensitivity. In a meta-analysis, an ADD-RS of 0 with D-dimer < 500 ng/mL provided high sensitivity for ruling out dissection (99.9%) (19). Hence, there is good justification to use a low ADD-RS and low D-dimer as a rule-out strategy. Expanding low-risk to include patients with an ADD-RS of ≤1 and negative D-dimer, compared to an ADD-RS of 0, resulted in improved specificity from 18.2% to 57.3% with minimal reduction in sensitivity, from 99.6% to 98.8% (20).

A meta-analysis in 2024 highlighted the trade-off between sensitivity and specificity when altering the ADD-RS threshold value to determine who should undergo advanced imaging. A lower ADD-RS threshold of >0 resulted in high sensitivity but low specificity whilst a higher ADD-RS threshold of >1 resulted in lower sensitivity but improved specificity (21). Resultingly, clinicians are in the unenviable position of balancing a missed diagnosis or over-investigating. As the potential consequences of an AAD misdiagnosis are life-threatening, there is a tendency to apply the strategy of high sensitivity. However, in this low prevalent population, the reduced specificity results in many CTAs performed without AAD identified. This is supported by both an observational cohort study and a single-centre retrospective study, which reported only 4.3% and 2.7% of CTAs performed for suspected AAD were positive, respectively (3, 4).

Other DDSPs have been reported including the AORTAs score which has similar performance to ADD-RS when both incorporate D-dimer (22), and the Canadian Practice guideline which uses history, risk factors and examination findings to categorise into patients into low-, intermediate- and high-risk probability groups. Low-risk patients require no further testing, intermediate-risk patients proceed to a D-dimer whilst high-risk patients proceed to a CTA (23).

Despite the potential of DDSPs to improve AAD diagnosis, their uptake has been limited. Contributing factors include lack of awareness, perceived suboptimal performance, the varied presentation of AAD, and potential loss of autonomy (24–26). Additionally, human decision-making is subject to fatigue, limited experience, and bias, and clinicians may miss or be unable to detect subtle relationships. Artificial intelligence can address some of these limitations and offers the potential to assist humans in the early diagnosis of AAD.

## Machine learning: concepts and applications in healthcare

Approximately 30% of the world's data is generated by the healthcare industry (27) and health data is stored in structured formats (e.g., tabular data) or unstructured formats (e.g., text, images, ECGs). With the adoption of the electronic health record (EHR) there has been an explosion of unstructured health data, which now accounts for an estimated 80% (28, 29).

Machine learning (ML) is the “field of study that gives computers the ability to learn without being explicitly programmed” (30). It often uses artificial neural networks which simulate the human brain and contain input, hidden and output layers. As health data modalities have their own formats and characteristics (31), different ML architectures excel with specific modalities.

Deep learning (DL), a subset of ML, uses artificial neural networks containing multiple hidden layers enabling it to learn complex non-linear relationships in data. DL techniques automatically extract a characteristic or data point, known as features, making it particularly effective for image recognition and natural language processing (NLP) tasks (32). However, to do so effectively DL applications require significantly more computational resources and larger training datasets.

### Learning paradigms in machine learning

Machine learning approaches can be broadly categorised as supervised, unsupervised, self-supervised, and semi-supervised according to how models learn from labelled and unlabelled data. In healthcare, the most suitable architecture often depends on the data modality. Transformer-based models dominate text tasks, non-DL gradient boosting decision trees have traditionally been used for tabular data, although transformers are emerging as an alternative and CNNs remain the most established architecture for ECG and CXR analysis, with increasing interest in vision transformers (ViT).

As early AAD diagnosis relies on routinely available ECG, CXR, tabular, and potentially text data, understanding the strengths and limitations of these modality-specific approaches is important when considering future DL model development (Table 1).

**Table 1:** Deep learning modality-specific architectures relevant to early AAD diagnosis.

**Modality**	**Typical architectures**	**Strengths**	**Limitations**	**Representative Examples**	**Relevance to Early AAD**
Text (EHR)	Transformers	Captures contextual meaning and relationships	Large; requires fine-tuning	BioBERT (100), BioClinicalBERT (101)	Extracts features from clinical notes, Foundation models available
Tabular data	Transformers, Hybrid models	Strong performance; interpretable	Sensitive to missing data, preprocessing	TabTransformer (102), TabNet (103), AC2 (104)	Includes demographics, risk factors, labs, vitals, risk factors
ECG	CNN, Vision transformer, Hybrid models	Captures temporal signal features	Requires large datasets; interpretability	ResNet (57), HuBERT (105) WT-CNN (106)	Core modality for early AAD, Foundation models available
CXR	CNN, Vision transformer, Hybrid models	Strong image classification, interpretability	Limited sensitivity for subtle findings	ResNet (57) ECGFounder (59) SA-DenseNet121 (107) health record;	Core modality for early AAD Foundation models available

AAD, Acute aortic dissection; CXR, chest x-ray; ECG, Electrocardiogram; EHR, Electronic health record.

### Machine learning with multiple modalities

Clinicians collect and appraise data from multiple modalities when making diagnostic decisions. Multimodal AI potentially represents an advancement enabling the integration and analysis of diverse data modalities, capturing inter-modal and intra-modal relationships to provide a more comprehensive and nuanced assessment. DL multimodal fusion improves performance compared to unimodal models, with gains in accuracy of 1.2%–27.7% and AUROC of 0.02–0.16 (33). The encoder employed prior to fusion is often dictated by the data modality used, with CNNs dominating images and RNNs or transformers for non-image data (34).

Again, domain specific adaptation improves performance. Med-Flamingo, the first medical specific large multimodal model (LMM) combined a ViT encoder with a LLM decoder, demonstrating a 20% performance improvement in medical visual question and answering tasks (35).

Encoder-only LMMs are typically based on BERT, processing and integrating image and text data, attempting to understand both the intra-modal and inter-modal relationships using attention. There are several medical specific encoder-only LMMs although training has been hampered by large image and text pair requirements. MedCLIP overcomes this issue by applying a novel decoupling approach achieving state-of-the-art performance in classification tasks (36).

### Multimodal fusion techniques

Multimodal fusion strategies have been categorised as early, intermediate or late based on the stage at which modalities are combined. With the emergence of DL, there has been a shift towards describing the type of fusion technique applied (37, 38).

Early fusion integrates raw data from each modality before entering a single model, enabling detection of inter-modal relationships but requires high computational demand due to heterogeneous, high dimensional data inputs (39). Intermediate fusion processes each modality independently, before fusing the learned feature representation, improving feature representation and identification of intra- and inter-modal relationships (33). Late fusion combines predictions from the independently processed models, often using simple or weighted averaging to make the final prediction (40). Whilst it cannot learn inter-modal relationships, late fusion has outperformed unimodal models (41) and early and intermediate fused models in classification tasks (42).

Considering fusion techniques, operation-based fusion combines features by concatenation, element-wise summation or multiplication, often requiring vectors with similar dimensions (38). Attention-based fusion focuses on the most relevant features from each modality. By adaptively weighting the significance of features both within and across modalities, attention-based fusion can result in improved accuracy and predictive performance. Various architectures are available based on the timing of intra-modality self-attention and inter-modality cross-attention. (37, 43) used cross-attention to fuse text, CXR and tabular data achieving superior multi-class disease classification compared to unimodal, early fusion, and late fusion methods. Tensor-based fusion combines multimodal data by constructing a multi-dimensional array, where each dimension represents a modality to capture inter-modal and intra-modal relationships. However, this approach has high computational demands. Hence careful consideration of feature and dimensionality is required (44). Graph-based fusion combines data modalities although implementation is complex. Recently, state-of-the-art-performance prediction metrics were achieved with domain specific auto-encoders employed before graph neural network (GNN) representation learning (45).

Hybrid DL fusion techniques have also demonstrate performance advantages. (46) used a transformer for text, CNN for images and shallow neural network for tabular data, prior to bimodal fusion with self- and cross-attention, and low-rank multimodal fusion forming a trimodal model. This model addressed missing data and achieved superior classification performance.

### Machine learning in decision support pathways

Specialised software can analyse individual patient data with the intent to assist clinicians in their decision-making. When implemented at the point of care, these systems can improve adherence to guidelines and aid in result interpretation (47). Since AAD is frequently misdiagnosed and linked to increased morbidity and mortality, the integration of AI into clinical workflows has the potential to improve diagnostic accuracy and impact patient outcomes (48).

## Deep learning in early acute aortic dissection diagnosis

To review the evidence on the use of DL models for the early diagnosis of AAD, in August 2025, a literature search was conducted using Medline via Ovid, Google Scholar and the Cochrane library to identify relevant publications. Search terms included combinations of “aortic dissection”, “artificial intelligence”, “AI”, “chest x-ray”, “CXR”, “deep learning”, “diagnosis”, “electrocardiograph”, “ECG”, “EKG”, “machine learning” and “tabular data”. Studies focused on non-deep learning architectures, advanced imaging modalities and case reports were excluded. References of identified publications were screened to identify additional eligible studies.

Seven studies using ECG only, CXR only or a combination of ECG, CXR and/or tabular data to train a DL model for the early diagnosis of AAD were identified (49–55). No studies using tabular-only or text-only data within a DL model were identified. A qualitative synthesis of the seven studies was performed, focusing on performance, DL architectures, addressing class imbalance, calibration, and explainability (Table 2).

**Table 2:** Methods—DL applications in the early diagnosis of acute aortic dissection (methods).

**Study**	**Year**	** Modality**	**Model Architecture**	**Pretrained**	**Class Imbalance Handling**	**Optimizer**	**Hyperparameters**	**Augmentation**	**Validation Strategy**	**Calibration**	**Explainability**
Lee et al. (49)	2022	CXR	CNN (ResNet18)	No	Weighted random sampling;weighted cross-entropy loss; ensemble voting	Adam	Batch size 16	Flip, flop, rotation	5-fold CV + external validation	No	Grad-CAM
							LR 1e-4				
Liu et al. (50)	2021	ECG + CXR	CNN ECG12Net + DenseNet121 (late fusion)	Yes	Weighted cross-entropy loss	Adam	Batch size 32	ECG cropping; CXR crop + inversion	5-fold CV + external + prospective validation	No	Class activation mapping
							LR variable				
Kolossváry et al. (51)	2023	CXR + clinical	CNN: DenseNet121 (late fusion)	Yes	Not reported	Adam	Batch size 64	Flip, flop rotation, zoom, lighting	Internal split + external validation	Yes	Grad-CAM
							cnn_learner				
Zhou et al. (52)	2024	ECG	CNN: custom (ResNet-inspired)	No	Matched control; focal loss	Adam	Batch size 4	No	10-fold CV + train/test split (internal only)	No	Correlation heatmap (Spearman)
							LR 3e-5				
Arita et al. (53)	2024	ECG	CNN: custom (ResNet-inspired)	No	Weighted loss (x129)	Not reported	Batch size and LR not reported	No	5-fold CV (internal only)	No	Grad-CAM
Lin et al. (54)	2024	CXR	CNN: InceptionV3, VGG19	Yes	Not reported	Adam	Batch size 20	Yes	Train/test split (internal only)	No	Class activation mapping
							LR 1e-4				
Wang et al. (55)	2025	ECG + tabular	CNN: ResNet34 feature extractor	No	Not reported	Not reported	100 epochs; 28 features	No	5-fold CV + prospective validation	No	SHAP

CV, cross-validation; CXR, chest x-ray; ECG, electrocardiogram; SHAP, Shapley Additive Explanations; Grad-CAM, Gradient-weighted Class Activation Mapping; LR, learning.

### Performance

Across the seven studies, AUROC ranged from 0.82 (50) using a CXR-based model to 0.968 (55) using ECG-derived features intermediate-level fused with laboratory data model. However, direct comparison of performance is challenging due to the substantial differences in data modalities, class imbalance and validation techniques. For example, in (55), performance was achieved in a small, near-balanced cohort comparing AAD to AMI. Meanwhile (50), and (53) used chest pain cohorts with high class imbalance employing weighted cross-entropy and weighted loss balancing techniques respectively.

ECG-based models AUROC ranged from 0.855 to 0.97 and CXR-based models from 0.82 to 0.955. Studies using a multimodal approach observed a performance improvement in some studies, suggesting potential benefit of multimodal DL for the early diagnosis of AAD. Importantly, (51) observed AUROC decreased from 0.86 to 0.67 when the model was applied to an external hospital, improving to 0.74 following fine-tuning with local data, demonstrating that internal validation performance is insufficient (Table 3).

**Table 3:** Results—DL applications in the early diagnosis of acute aortic dissection.

**Study**	**Modality**	**Location**	**N (+ive AAD)**	**Validation**	**AUROC**	**Sens (%)**	**Spec (%)**	**NPV (%)**	**F1-score**	**PPV (%)**	**Accuracy**
Liu et al. (50)	ECG	Taiwan	45,806	Internal	0.863	75.8	83.0	99.8	–	3.2	–
Wang et al. (55)	ECG	China	277	Internal	0.855	88.4	81.4	–	0.82	–	84.9
	ECG + labs				0.968	96.9	96.7	–	0.97	–	96.7
Arita et al. (53)	ECG	Japan	19,170	Internal	0.936	85.7	90.4	-	0.13	–	91.7
Zhou et al. (52)	ECG	China	1,565	Internal	0.964	91.4	94.6	–	0.93	–	0.93
				(chest pain cohort)	0.928	83.9	90.3	–	0.87	–	87.1
Liu et al. (50)	CXR	Taiwan	43,365	Internal	0.825	71.1	77.8	99.8	–	1.4	–
Lin et al. (54)	CXR Inceptionv3	Taiwan	1,625	Internal	0.820	68.0	88.0	–	0.76	–	78.0
	VGG19				0.840	56.0	88.0	–	0.67	–	72.0
Lee et al. (49)	CXR	South Korea	3,331	External	0.955	94.4	88.7	97.8	0.84	75.0	90.2
Kolossváry et al. (51)	CXR + Clinical	USA	28,755	Internal (threshold)	0.860	99.0	17.3	98.8	0.36	22.0	32.8
	CXR + Clinical		22,764	External (threshold)	0.670	99.0	6.8	96.4	0.36	21.6	25.8
	CXR + Clinical (fine-tuned)		22,764	External (threshold)	0.740	99.0	10.4	96.6	0.37	22.7	29.0
Liu et al. (50)	ECG + CXR	Taiwan	40,110	Internal	0.882	76.7	81.7	99.9	–	1.7	–
	ECG + CXR + D-dimer		Subgroup	External	0.910	68.4	90.4	99.7	–	6.2	–

N, number; CXR, chest x-ray; ECG, electrocardiogram; AUROC, Area under the receiver operating curve; Sens, sensitivity; Spec, specificity; NPV, negative predictive value; PPV, positive predictive value.

### Architectures, foundation models and fusion techniques

All identified models employed CNN architectures with a notable absence of vision transformers, which may reflect their recent development (56). Moreover, no models were created using graphs. For ECG-based tasks, ResNet architectures dominated with all models employing either a ResNet or custom ResNet-inspired architecture. The residual (skip) connections in ResNets address vanishing gradient problem, enabling the use of deeper networks (57) to learn subtle ECG patterns across various layers such as the QRS, ST changes and cardiac rhythm, and have demonstrated benchmark level performance compared to other architectures (58).

For CXR tasks, no single architecture was employed. However, ResNet18 and DenseNet121 achieved strong performance. Again, the heterogeneity in training strategies and hyperparameters limits comparisons. The strong performance of the relatively lightweight ResNet18 suggests smaller model architectures may be sufficient for accurate classification and potentially useful in real-time scenarios using edge devices.

All studies either trained randomly initialised models from scratch on task specific data or fine-tuned using pretrained weights. No study employed a cardiac specific foundation model such as ECGFounder, which following fine-tuning outperforms task-specific ResNet architectures by 4%–6% in classification tasks (59). This highlights an important research gap.

Most multimodal studies employed late-level fusion, training separate unimodal models before combining their output probabilities, typically by logistic regression (50, 51). Some models applied clinical or tabular variables *post-hoc* (52, 53). The exception was (55), who employed intermediate-level fusion by combining ResNet derived ECG embedding vectors with tabular laboratory features before passing them to a classifier (55). Whilst this model achieved the highest discrimination performance, potentially indicating the advantage of intermediate-level fusion, this should be interpreted with caution, given the classification task (AAD vs. AMI) with a small, near-balanced training dataset.

### Approaches to class imbalance

Measures to address class imbalance also varied across studies and were often modality dependent. For ECG-based tasks, one study augmented data by ECG-cropping (50), while this and other studies used loss-based approaches including weighted cross-entropy loss (50), focal loss (52), and class-weighted loss functions with upweighting of the AAD class (53). For CXR-based tasks, studies primarily used simple image augmentation techniques including flipping, rotation, cropping and resizing (49–51), and one study used weighted cross-entropy loss (50). No study employed more advanced augmentation techniques such as generative adversarial networks (GAN) or signal-level ECG data augmentation e.g., amplitude scaling, addition of Gaussian noise or time warping, highlighting another research gap.

### Internal vs. external validation

Two studies applied geographic external validation. (51) evaluated their multimodal model trained at Massachusetts General Hospital on an external dataset from Brigham and Woman's Hospital, and observed a performance reduction that partially recovered following fine-tuning. (49) reported validation with an AUROC of 0.955, however the AUROC with internal validation was not reported. (50) performed temporal validation, using separate patient cohorts at the same institution. The remaining four studies relied on internal validation, which can result in optimistic performance estimates.

### Calibration

Discrimination reflects a model's ability to distinguish between patients with and without the outcome, but does not assess the accuracy of predicted probabilities, whereas calibration measures the agreement between predicted and observed outcomes. A poorly calibrated model may demonstrate high discrimination but produce inaccurate risk estimates that may misinform clinical decision making (60). Well calibrated models are essential in safety critical applications such as healthcare, particularly in life-threatening conditions such as AAD. Importantly, in neural networks, calibration decreases with increasing depth and width of ML architectures, class imbalance, choice of loss function and optimisation strategy, highlighting the importance of calibration in DL models (61).

Calibration was reported in only one study. (51) applied Platt scaling to the DL model outputs and assessed calibration using plots, observing good agreement at lower predicted risk levels but reduced calibration at higher risk probabilities. At a 99% sensitivity threshold, the DL model deferred approximately seven times more patients from further testing compared to the clinical model.

### Explainability

Most models incorporated some form of explainable AI (XAI), with gradient-weighted class activation mapping (Grad-CAM) or class activation mapping (CAM) most frequently applied. With CXR-based models (51), and (54) used Grad-CAM to demonstrate predictions were driven by anatomically plausible regions, with the latter also showing the abnormalities on CXR correlated to the diseased area on CTA. (50) applied CAM to CXR and ECG modalities, observing with CXRs, the model attended to the mediastinum structures, while the ECG model focused on ST-segment and T-wave morphology. They also observed ST-elevation was treated as a non-AAD feature by the model suggesting the model may also be learning to predict non-AAD rather than identify AAD. This may have implications when the model is applied to datasets with different class balances. With the ECG-based only models, (53) applied Grad-CAM, observing lead V1, QRS and ST-T wave segments were most strongly weighted. (55) used SHAP values in the tabular dataset and ECG embeddings, demonstrating ECG-derived features were of greater importance to model prediction than D-dimer.

A limitation with XAI applied to CXR and to some extent ECGs, is that current techniques provide qualitative spatial or saliency maps, that support plausibility, but they do not provide causal explanation. Finally, no study assessed if the application of XAI altered diagnostic accuracy or clinical decision-making.

In summary, DL architectures applied to ECG and CXR data demonstrate strong discriminatory performance. However, several limitations constrain this conclusion, including the limited number of studies, some with highly selective cohorts and from restricted local datasets. The reduction in performance observed during external validation underscores the importance of assessing model generalisability. The high discriminatory performance reported when intermediate-level fusion is used highlights the need for further research in early and intermediate-level fusion. The limited use of calibration, despite its critical importance in healthcare decision-making, represents a notable gap in the current literature. Whilst several studies included tabular data like D-dimer and demographic information, this was using non-DL approaches. Currently, there is no published research into the role of DL with tabular data or text in the early diagnosis of AAD.

## Deep learning models for aortic dissection: development and implementation challenges

### Data scarcity

The power of DL lies in training from large datasets. When using small datasets, DL models are prone to overfitting, a process where the model learns the data too specifically, performing well with the training data but poorly with test data. AAD is rare and access to large healthcare datasets even when deidentified is often restricted to maintain privacy, confidentiality and jurisdictional regulations. A challenge of applying DL techniques for AAD diagnosis is identifying and accessing large multimodal healthcare datasets with high numbers of AAD cases.

Federated learning (FL) offers a potential solution for data scarcity by enabling model training across multiple decentralised sites without the need to share raw patient data, instead aggregating model weights. This approach can reduce data access limitations and enhance data heterogeneity. In CXR-based classification tasks, federated learning models have demonstrated modest performance improvements compared to models trained on single-site data, likely reflecting increased sample size. Whilst performance is generally comparable to centrally trained models, FL approaches have shown improved external validation performance, suggesting benefits from data heterogeneity (62).

With FL, trust between collaborators is required and recent work has focused on improving model reliability and privacy preservation. For example, a FL approach applied to ECG data within a digital twin framework incorporated noise injection to reduce the risk of patient data leakage, achieving a low data leakage rate (63). Work in the consumer domain has proposed a trust-oriented framework (TOFL-IoT) incorporating a governance layer and multi-level trust evaluation across devices, network and model components, dynamically aggregating its outputs to strengthen the reliability of the global model (64). Whilst not evaluated in healthcare settings, such an approach highlights important considerations for maintaining trust and reliability in future multicentre AAD model development.

### Missing data in EHR datasets

Electronic Health Records contain missing data, which can result in biased ML models with suboptimal performance. Approaches to deal with missing data include deletion and imputation, although deletion can introduce bias, particularly when the reason for the missingness is not random (65). Imputation, the process of replacing missing values with estimated values, preserves data and can complete datasets. Statistical imputation methods include mean value, multiple imputation by chained equations and expectation-maximisation algorithms. More sophisticated approaches include the ML technique K-Nearest Neighbour and DL techniques such as Generative Adversarial Networks (GAN) and autoencoders (66). GANs use a generator to create synthetic data, and a discriminator attempts to distinguish between the real and synthetic data. Through iteration the model improves until the synthetic data is indistinguishable from the real data. However, they are constrained by significant computational demands and prolonged sampling times (67). For CXR synthesis, GANs and hybrid GANs have demonstrated superior performance with less computation time compared to autoencoders (68). For ECG synthesis, variational autoencoders excel with training speed, diversity and model size whilst GANs achieve higher fidelity (68–70). In tabular data, a GAN with semi-supervised learning improved a DL models predictive performance (71).

Large Language Models have proven useful in synthetic medical text generation, with GPT-2 outperforming a GAN and RNN in generating synthetic healthcare text, and subsequent models trained with real and synthetic data outperformed those with real data (72). GPT-4 has created tabular data with accurate statistical properties (73). However, generative LLMs can hallucinate, resulting in inaccuracies, and there are no standardised metrics to evaluate the fidelity and accuracy of LLM generated synthetic data (74).

### Class imbalance

Acute aortic pathologies account for 0.2% of chest pain presentations to the ED via ambulance with ischaemic heart disease the most common specific cause (14.9%) and non-specific pain the largest group (46%) (75). This class imbalance results in ML bias towards the majority, non-AAD cases.

Several techniques are available to address class imbalance. Data-level techniques include under-sampling non-AD cases but can results in loss of data (76). Adaptive Synthetic Sampling (ADASYN), an oversampling technique, generates synthetic minority class data that are more challenging to learn, improving class imbalance and shifting the classification boundary towards harder cases, which may enhance model performance (77). Synthetic Minority Over-Sampling Technique (SMOTE) creates synthetic data using a k-nearest neighbour algorithm, demonstrating improved performance when applied to imbalanced tabular data in DL classification tasks (78).

Deep Learning models, including variational autoencoders and GANs can improve class imbalance and model performance through data augmentation (71), although their role in early AAD diagnosis has not been explored.

Feature selection, a statistical based algorithm method, can bias towards the majority class whilst decision-tree and wrapper methods can increase emphasis on features favouring the minority class. Cost-sensitive learning addresses imbalance by assigning different costs to misclassifications, improving class imbalance without changing the data distribution, although can increase overfitting of the minority class data (79). Ensemble methods can partially mitigate class imbalance through combining multiple individual classifiers. Techniques include bagging, boosting and stacking.

Lastly, hybrid models have demonstrated value in addressing AAD class imbalance. In a tabular dataset (80) found SmoteBagging (SMOTE plus bagging) improved recall. A subsequent hybrid model incorporating under-sampling, feature selection, cost-sensitive learning and bagging further improved performance (81).

### Evaluation metrics for DL model assessment in the presence of class imbalance

When class imbalance exists, careful selection and interpretation of evaluation metrics is warranted as diagnostic accuracy can mislead. The model could misclassify all AADs but result in an accuracy >99%, provided all non-AADs are correctly classified.

Precision, the proportion of true positive predictions among all positive predictions, focusing on minimising false positives. Recall (sensitivity), is the proportion of true positive cases identified from all ground truth positive cases, focusing on minimising false negatives (82). F1-score, the harmonic mean of precision and recall gives equal weights to both metrics therefore considers both false positives and false negatives (83). In AAD classification, where high-class imbalance exists and false negatives are particularly important, the F1-score provides a more nuanced assessment of a model's performance than accuracy.

The receiver operator characteristic curve (ROC) evaluates a models ability to balance the true positive rate against the false positive rate. By varying the threshold for positive classification, a curve is plotted illustrating this trade-off. A 45° line indicates random performance, while curves approaching the top left corner indicates high discriminatory performance. The AUROC provides a measure of the overall model's ability to differentiate between groups. Although interpretation varies, AUROC >0.9 are generally considered excellent, >0.8 good, and <0.6 poor (84).

The precision-recall curve (PRC) plots positive predictive value against sensitivity and evaluates the model's capacity to identify true positives, in our case AAD, the minority class, whilst disregarding the identification of the healthy majority class. The area under the precision-recall curve (AUPRC) provides an overall measure of the model's performance and is generally regarded, though not universally, as more suitable in the presence of high-class imbalance (85).

### Explainability and trust in DL models

Healthcare decision making requires accuracy, reliability, and accountability with significant consequences when errors occur. The multi-layered hidden “black box” architectures of DL models limit explainability (86). Clinicians require a plausible explanation and understanding before adopting into practice, with a lack of explainability reducing uptake (87). Explainable AI (XAI) aims to bridge the gap between ML models and human understanding, to provide transparent and interpretable insights into how models generate predictions. In emergency medicine, XAI techniques demonstrate clinical predictors which correlate with well-established medical knowledge (88).

The European Union Artificial Intelligence Act requires AI-based healthcare decision support systems be intrinsically explainable or employ *post-hoc* methods (89). Intrinsic XAI methods are integrated during model development, providing explanations as part of its output, whilst *post hoc* XAI methods analyse trained models without altering its structure.

Post-hoc methods included SHapley Additive exPlanations (SHAP) which quantify the marginal contribution of each feature relative to the other features (90). It can be computationally expensive, with multiple versions available depending on model architecture and data modality and was applied by (55) on ECG embeddings in AAD diagnosis. Gradient weighted saliency considers the gradient loss through back-propagation to quantify feature importance to the final prediction. Gradient-weighted class activation maps (Grad-CAM) produce heat maps highlighting regions in images of importance in decision making, providing a visual explanation, inducing plausibility if the area highlighted corresponds to the known pathology (91). Recently, a gradient weighted saliency method called Attention Guided CAM extends this approach to ViTs, combining gradient information with self-attention scores, outperforming existing ViT explainable methods (92).

Local Interpretable Model-agnostic Explanations (LIME) explain individual predictions by approximating them with a simpler, interpretable surrogate, such as linear model or decision tree. LIME alters the input data near a prediction to assess how the model output changes, identifying the most influential features for the prediction (93).

## Future research directions

### Model architecture, performance and generalisability

Across the included studies, model performance was moderate and below thresholds required for clinical diagnostic support, indicating that further research advances are needed. Importantly, all models were based on CNN architectures, with a notable absence of transformer-based architectures despite their demonstrated superior performance in other medical domains, highlighting an important research gap. For example, transformer-based architectures such as BioClinicalBERT has achieved state-of-the-art performance healthcare NLP (94), ViT HeartBEiT outperformed CNNs in ECG classification (95), and TabNet may provide performance improvements for tabular data.

Given the reported misdiagnosis rate of AAD is 33.7%, even moderate DL performance is promising. However, these comparisons require cautious interpretation as most models are retrospective, lack calibration or real-world validation. Most studies used single-centre datasets raising concerns of generalisability. The reduction in performance observed with external compared with internal validation, observed by (51), highlights the impact of geographic and institutional variation, including dataset shift from differences in patient populations, available technologies and clinical workflows. Widespread external validation remains essential in demonstrating generalisability and transportability prior to clinical implementation.

Additionally, model performance requires evaluation in real-time clinical settings to assess inference latency, integration with the EHR and edge devices, and clinician interaction. Ultimately, models need to demonstrate clinical benefit, with improvements in misdiagnosis rates, time to diagnosis, morbidity and mortality.

### Multimodal DL modelling provides a key opportunity

Multimodal DL models, integrating text, ECG, CXR and tabular data offer the potential to improve diagnostic performance. However, multimodal DL studies in AAD, with the exception of (55), employed late-level fusion with minimal performance enhancement. Encouragingly (55), demonstrated the highest discrimination performance using intermediate-level fusion, albeit with dataset- and task-specific caveats. Intermediate-level attention-based fusion captures intra- and inter-modal relationships using self- and cross-attention, and has demonstrated performance gains in predicting Alzheimer's disease progression (96). Applying this architecture in AAD may represent a key opportunity to improve early diagnostic accuracy.

### Data scarcity and class imbalance: the role of augmentation and federated learning

Aortic dissection is rare, and access to large, labelled multicentre datasets for training DL models remains a significant limitation. Federated learning represents a promising strategy to address data scarcity and heterogeneity, to improve model performance whilst maintaining patient privacy and meeting jurisdictional regulations. This approach may be particularly valuable in AAD, where individual centres are unlikely to accumulate sufficient case numbers independently.

Data augmentation to address class imbalance, beyond the basic augmentation and loss weighting strategies implemented in existing DL models is warranted. GANs and variational autoencoders have synthesised CXRs and ECGs, and their role in AAD diagnosis AAD to both augment data and address missing data in multimodal training sets, warrants further investigation.

### Evaluation metrics and calibration

Evaluation metrics need to account for residual class imbalance inherent to AAD, to provide better insight into model performance. AUROC and F1-score reflect discrimination but do not assess either accuracy of predicted probabilities or clinical utility.

Despite calibration's critical role in healthcare settings, where accurate probability estimates are required to support clinical decision making, only one DL study incorporated calibration into model evaluation, and this represents a significant research gap. Calibration directly influences threshold selection and given the consequences of a missed AAD are severe, diagnostic strategies should prioritise high sensitivity and negative predictive value. Future work should include *post-hoc* calibration techniques and evaluation strategies using clinically relevant thresholds such as sensitivity ≥99% or a high rule-out threshold, to minimise misdiagnosis.

Additionally, causal inference frameworks are required to isolate the effect of the AI decision support system from confounding factors and ensure systems better predict and support interventions that improve outcomes.

### Clinical implementation and real-world evaluation

Model training is computationally intensive, typically requiring high-performance GPUs. Inference is often less resource demanding and faster, making real-time clinical decision support on edge devices feasible. For ECG-based models, 1D-signal representations outperform 2D image-based representations, while avoiding the additional computational burden of signal to image transformation (97). These 1D-models are compatibility with commercial digital ECG systems but limits the use of smartphone acquired 2D ECG photos. CXR-based models use 2D image data, which may allow smartphone derived photo inputs, but require emergency departments and their institutions to have sufficient information technology infrastructure and human resource support. When selecting model architecture and data inputs for AAD diagnosis, the available clinical computational resources needs to be considered. Doing so maximises the prospect of clinical deployment.

Following the introduction of a DL-assisted decision support system, its impact on clinical behaviour requires monitoring and assessment, with the potential for automation bias, as clinicians become over-reliant on or place excess trust in support system outputs at the expense of critical thinking, as has been observed in other areas (98).

### Safety, ethics and explainability

As AI systems integrate into healthcare, their deployment must be guided by the principles of responsible AI. This entails ensuring safety, trustworthiness, and ethical integrity throughout the design and implementation process. They must be designed with inclusivity in mind and implemented with fairness to avoid exacerbating existing healthcare disparities. This model development needs to consider the various information technology infrastructures across the world, otherwise there is potential to exacerbate disparities. Given the sensitivity of health data, safeguarding patient privacy through secure data storage and controlled access protocols are essential to uphold ethical principles and maintain trust (99).

We need to better understand how models come to their predictions. What features and what modality interactions were relevant to decision making. Both the integration of XAI techniques and appraisal of their performance in the early diagnosis of AAD is essential. This knowledge can inform the technical development and implementation in clinical practice, improve clinician trust and utilisation, and comply with legislative requirements.

## Discussion

Acute aortic dissection remains a diagnostic challenge, with a high misdiagnosis rate, low diagnostic yield of advanced imaging, and only moderate discriminatory performance of current DDSPs. DL excels at identifying complex relationships within large datasets and leveraging this capability may improve early decision making in AAD, optimally improving diagnostic efficiency and clinical outcomes.

Existing DL research for early AAD diagnosis is limited, with most studies focusing on CNN architectures applied to CXR or ECG data and demonstrating only moderate discriminatory performance. Studies are largely retrospective, use single centre datasets, and with heterogeneous methodologies, restricting comparisons and generalisability. The absence of transformer architectures, which have demonstrated state-of-the-art performance when trained with text, ECG and CXRs in other healthcare domains is notable. More advanced early- and intermediate-level fusion techniques requires further investigation, and the observed reduction in performance with external validation underscores the importance of demonstrating generalisability and transportability. Calibration, which is critical to healthcare decision making was rarely assessed, limiting confidence in predicted risk estimates.

From a practical perspective, several factors limit the clinical applicability of DL models for the early diagnosis of AAD. Widespread external validation remains essential prior to clinical implementation. Moreover, model performance requires evaluation in real-time clinical settings to assess inference latency, integration with the EHR and edge devices, and clinician interaction. Ultimately, models need to demonstrate clinical benefit, with reductions in misdiagnosis rates, time to diagnosis, morbidity and mortality. Addressing these gaps will be essential to transition DL models for AAD from the bench to clinically deployable decision support tools.
